# CD8TCEI-EukPath: A Novel Predictor to Rapidly Identify CD8^+^ T-Cell Epitopes of Eukaryotic Pathogens Using a Hybrid Feature Selection Approach

**DOI:** 10.3389/fgene.2022.935989

**Published:** 2022-07-22

**Authors:** Rui-Si Hu, Jin Wu, Lichao Zhang, Xun Zhou, Ying Zhang

**Affiliations:** ^1^ Yangtze Delta Region Institute, University of Electronic Science and Technology of China, Quzhou, China; ^2^ School of Management, Shenzhen Polytechnic, Shenzhen, China; ^3^ School of Intelligent Manufacturing and Equipment, Shenzhen Institute of Information Technology, Shenzhen, China; ^4^ Beidahuang Industry Group General Hospital, Harbin, China; ^5^ Department of Anesthesiology, Hospital (T.C.M) Affiliated of Southwest Medical University, Luzhou, China

**Keywords:** eukaryotic pathogens, T-cell epitopes, machine learning, hybrid features, LightGBM

## Abstract

Computational prediction to screen potential vaccine candidates has been proven to be a reliable way to provide guarantees for vaccine discovery in infectious diseases. As an important class of organisms causing infectious diseases, pathogenic eukaryotes (such as parasitic protozoans) have evolved the ability to colonize a wide range of hosts, including humans and animals; meanwhile, protective vaccines are urgently needed. Inspired by the immunological idea that pathogen-derived epitopes are able to mediate the CD8^+^ T-cell-related host adaptive immune response and with the available positive and negative CD8^+^ T-cell epitopes (TCEs), we proposed a novel predictor called CD8TCEI-EukPath to detect CD8^+^ TCEs of eukaryotic pathogens. Our method integrated multiple amino acid sequence-based hybrid features, employed a well-established feature selection technique, and eventually built an efficient machine learning classifier to differentiate CD8^+^ TCEs from non-CD8^+^ TCEs. Based on the feature selection results, 520 optimal hybrid features were used for modeling by utilizing the LightGBM algorithm. CD8TCEI-EukPath achieved impressive performance, with an accuracy of 79.255% in ten-fold cross-validation and an accuracy of 78.169% in the independent test. Collectively, CD8TCEI-EukPath will contribute to rapidly screening epitope-based vaccine candidates, particularly from large peptide-coding datasets. To conduct the prediction of CD8^+^ TCEs conveniently, an online web server is freely accessible (http://lab.malab.cn/∼hrs/CD8TCEI-EukPath/).

## Introduction

Pathogen-derived antigen epitopes displayed on the surface of host antigen-presenting cells can be presented by major histocompatibility complex (MHC) molecules (also called human leukocyte antigen in humans) to the different subsets of T cells. Typically, MHC-I molecules present relatively fixed peptide lengths (usually 8–11 residues) to CD8^+^ T cells, thereby activating cytotoxic T lymphocytes to destroy invading pathogens (Trolle et al., 2016), whereas MHC-II molecules with an open peptide-binding groove have the ability to recognize peptides of highly variable lengths (usually 9–22 residues) that activate CD4^+^ helper or regulatory T cells (Holland et al., 2013). Obviously, antigen epitopes that trigger CD8^+^ T cells or CD4^+^ T cells bear essential differences during the process of host adaptive immune responses. Therefore, identifying what pathogen peptides will be presented to specific T cells is critical information for understanding infectious etiologies, developing diagnostic assays, and designing epitope-based vaccines against infectious agents.

Conventional approaches for T-cell epitope identification have depended entirely upon experimental technologies and experiences and are obviously time-consuming and costly. As a result, alternative computational approaches to implement antigen epitope identification have become powerful methods in immunology and vaccinology research and have significantly decreased the experimental load associated with epitope identification (Brusic et al., 2004; Zhang et al., 2012). To date, most T-cell epitope prediction tools have been developed using machine learning algorithms to train various experimental data, which are generally available in specialized epitope databases, such as the Immune Epitope Database (IEDB) (Vita et al., 2019). Since the first computational approach for epitope prediction was introduced more than 30 years (Sette et al., 1989), the performance of prediction methods in recent years has obtained significant advancement with the accumulation of positive epitope data, the development of machine learning algorithms, and the reduction of computational cost. These advancements are seen in the development of machine learning models to identify T-cell epitopes in various infectious agents, including pathogenic prokaryotes (such as bacteria) (Pamer et al., 1991; Nagpal et al., 2018; Zadeh Hosseingholi et al., 2020), viruses (Bukhari et al., 2021; Sharma et al., 2021; Xu et al., 2021), and pathogenic eukaryotes (such as parasitic protozoans) (Goodswen et al., 2014; Goodswen et al., 2021).

Among infectious agents, eukaryotic pathogens have evolved into several distinct phylogenetic lineages and bear resourceful abilities to affect a wide range of hosts, including humans and animals, resulting in significant effects on the aspects of global public health and considerable economic loss to the agricultural community (Haldar et al., 2006). Since a high level of MHC polymorphism in infected hosts and a large number of unknown functional proteins exist in eukaryotic pathogens (Hu et al., 2022), this undoubtedly produces challenges for T-cell epitope identification. Although presently some available software systems for *in silico* T-cell epitope prediction have been developed, including the NetCTL server (Larsen et al., 2005), the NetMHCpan server (Jurtz et al., 2017), and the MHCflurry server (O’Donnell et al., 2018), there is no guarantee that all these tools produce good quality predictions (Resende et al., 2012; Bordbar et al., 2020; Zawawi et al., 2020). Moreover, a general analysis of MHC-peptide binding prediction, overlooking specific patterns of MHC-presented peptides recognized by different types of T-cell receptors, may lead to lower predictive accuracy.

Given the wealth of state-of-the-art machine learning algorithms available and public experimental data, it is necessary to keep comparing the performance of different methods reciprocally and develop effective tools for the identification of T-cell epitopes in pathogen biology research. In the present study, based on MHC-I T-cell peptides collected from the IEDB database and experimentally validated neoantigen epitopes available from previous Review articles, we developed a novel machine learning-based method to identify CD8^+^ T-cell immunogenic epitopes in eukaryotic pathogens. Our method adopted the best hybrid feature descriptor and classifier to establish a prediction model and finally achieved an accuracy of 79.255% in ten-fold cross-validation and an accuracy of 78.169% in the independent test. Finally, a user-friendly web server named CD8TCEI-EukPath was developed, which will be helpful for scientists to rapidly screen epitope-based vaccine candidates from a plethora of mass spectrometry peptidome data.

## Methods and Materials

### Dataset Preparation

Eukaryotic pathogens (Eukpaths), such as protozoans and fungi, are important causative agents that cause serious infectious diseases in humans and animals; however, there is a lack of systematic collection of Eukpath-derived antigenic epitopes associated with the host immune response. Additionally, many previous works have pointed out that stringent datasets are considered important for the performance of a predictive model. In particular, peptide sequences for most T-cell epitopes (TCEs) usually have a short length, which easily leads to biased estimates if peptide sequences in a dataset have high similarity.

The present study collected datasets concerning positive and negative CD8^+^ TCEs available from the IEDB database (http://www.iedb.org/), following the search strategy: Eukaryote T-cell and class I MHC restriction (accessed on 15 October 2021). After data processing, 809 TCEs and 1,715 non-TCEs for Eukpaths were retained as positive and negative datasets, respectively. A detailed description of Eukpaths is included in Supplementary Table S1. In addition, we also obtained 371 experimentally determined peptide sequences for host CD8^+^ T cells that are described in the latest Review articles, in which have gave a detailed list regarding peptide sequences in three important parasites [i.e., *Plasmodium falciparum* (Heide et al., 2019), *Toxoplasma gondii* (Javadi Mamaghani et al., 2019), and *Trypanosoma cruzi* (Ferragut et al., 2021)] and, eventually, a total of 1,180 TCEs were reorganized as the positive dataset.

Before dividing the positive and negative datasets into training and testing sets, we performed data preprocessing, such as removing repeat sequences and sequences with high sequence identity. Repeat peptide sequences in the positive sample were removed using SeqKit software (Shen et al., 2016), and peptide sequences in positive and negative samples with more than 90% sequence identity were removed using the CD-HIT Suite server (Huang et al., 2010). Finally, a total of 706 TCEs from the positive dataset were retained, and an equal number of non-TCEs were randomly selected from negative datasets.

Regarding machine learning, training datasets are used to train a predictive model, and based on evaluation through testing sets, an optimal classifier was selected. We randomly selected 80% of datasets from both positive and negative samples as training sets and the remaining 20% as testing sets. Note that the training and testing sets can be downloaded from http://lab.malab.cn/∼hrs/CD8TCEI-EukPath/download.html.

### An Overview of the Established Predictor

**FIGURE 1:**
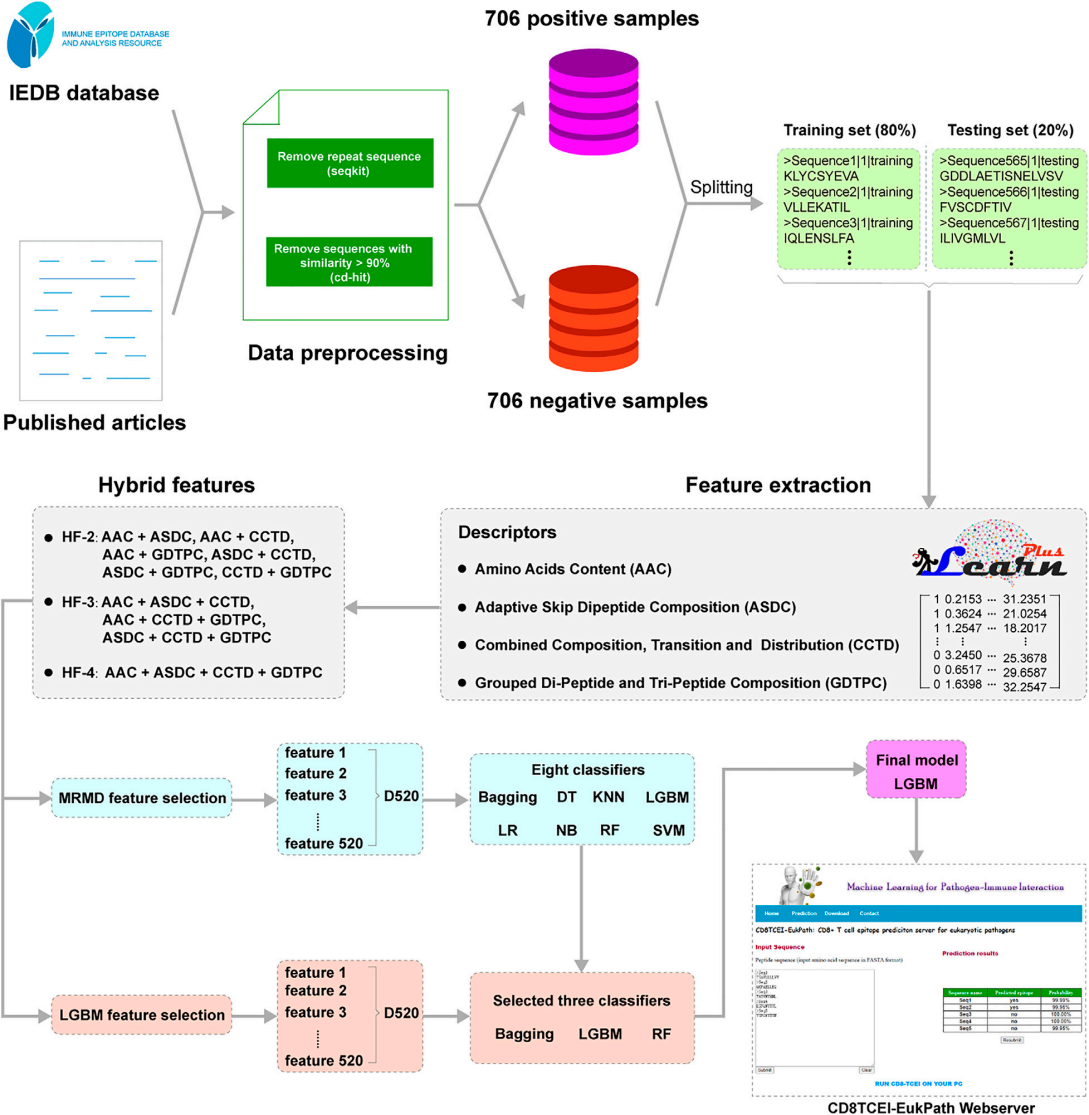
A technology roadmap of the machine learning model proposed in this study.

A modeling overview of the proposed approach is illustrated in Figure 1. CD8TCEI-EukPath allows users to utilize a large volume of peptide sequences, such as peptide-coding datasets available from mass spectrometry peptidomics, to serve as input sequences. First, each sequence is subjected to the feature representation based on the proposed hybrid feature scheme. Regarding machine learning modeling, hybrid feature identification is a useful approach for improving prediction performance and has been extensively applied to the identification of specific peptide sequences, such as anticancer T-cell antigen epitopes (Wei et al., 2018; Beltran Lissabet et al., 2019; Charoenkwan et al., 2020; Jiao et al., 2021). The detailed hybrid feature representation method can be seen in the subsequent Section 2.3. Then, hybrid features for each sequence are transmitted to the well-trained prediction model. In the final evaluation of the models, we choose the LightGBM (LGBM) classifier as the optimal training model. Eventually, the LGBM-based model will give an estimated score in the prediction results to differentiate TCEs from non-TCEs. If the prediction possibility of more than 50% is considered to indicate the true TCE and lower values indicate non-TCEs, the prediction possibility is calculated with a range from 0 to 100%.

### Feature Extraction

Protein or peptide sequences are composed of amino acids. In the standard amino acid alphabet, 20 different amino acids can be represented as {A, C, D, E, F, G, H, I, K, L, M, N, P, Q, R, S, T, V, W, and Y}. To establish a machine learning model, an essential step is to extract amino acid features from protein or peptide sequences, typically regarding structural and physicochemical properties of amino acids through a transformation from sequence to numerical vector (Chen et al., 2018; Chen et al., 2020; Chen et al., 2021). In this study, the iLearnPlus platform (Chen et al., 2021) was used to conduct feature extraction of the peptide sequence, as described below.

1) Amino Acid Composition (AAC). AAC is a commonly used descriptor that has been successfully applied to protein classification and anticancer peptide prediction (Bhasin and Raghava, 2004; Wei et al., 2018; Jiao et al., 2021). AAC is encoded based on calculating the occurrence frequency of each amino acid in a peptide sequence. The frequencies of AAC can be calculated as follows:

where N(i) represents the number of amino acid type *i*, L represents the length of a peptide sequence, and f(i) is the calculated composition frequency for a specific amino acid type *i*.

2) Adaptive Skip Dinucleotide Composition (ASDC). The ASDC descriptor is a modified dipeptide composition proposed by Wei et al., 2017a and Wei et al., 2017b. This descriptor has the advantage that it not only fully considers the relevant information between adjacent residues but also considers the intermediate residue. For a given protein or peptide sequence, this descriptor can generate 400-dimensional feature vectors (*fv*) that are presented by:

ASDC=(fv1, fv2, fv3,…,fv400)

$$fvi=\frac{\sum_{k=1}^{M-1}N_{i}^{k}}{\sum_{i=1}^{400}\sum_{k=1}^{M-1}N_{i}^{k}}$$

$f\left(i\right)=\frac{N\left(i\right)}{L},\;i\in$ {A, C, D…Y}

In the formula, *fvi* indicates the occurrence frequency of all possible dipeptide pairs with ≤ *M−1* intervening amino acids.

3) Combined Composition, Transition, and Distribution (CCTD). The CCTD features represent a global description of amino acids’ structural or physicochemical attributes, such as hydrophobicity, normalized van der Waals volume, polarity, polarizability, charge, secondary structures, and solvent accessibility of a peptide sequence (Dubchak et al., 1995; Tomii and Kanehisa, 1996; Dubchak et al., 1999). The CCTD contains three descriptors, namely, composition (C), transition (T), and distribution (D).
• Composition: The composition descriptor computes the percentage frequency of polar (RKEDQN), neutral (GASTPHY), and hydrophobic (CLVIMFW) residues in a given peptide sequence. It can be calculated as follows:

$$C=\;\frac{N\left(i\right)}{L},\;i\in \left\{polar,\;neutral,\;or\;hydrophobic\;residues\right\}$$

*N (i)* represents the number of amino acid type *i* in the encoded sequence*,* and *L* represents the length of the peptide sequence.

• Transition: The transition descriptor indicates the percentage frequency of amino acids that transition between the three groups, i.e., polar, neutral, and hydrophobic groups. The formula can be defined as follows:

$$T\left(i,j\right)=\frac{N\left(i,j\right)+N\left(j,i\right)}{L-1}$$

 i,j∈ {(polar, neutral), (neutral, hydrophobic), (hydrophobic,polar)}

where N(i,j) and N(j,i) represent the number of dipeptides that appeared in ‘*i, j*’ and ‘*j, i*’, respectively, and *L* represents the length of a peptide sequence.

• Distribution: The distribution descriptor describes the distribution of amino acids for each of the three groups (polar, neutral, and hydrophobic) in the sequence. There are five descriptor values for each group, and they are the corresponding position fractions in the entire sequence concerning first residues, 25% residues, 50% residues, 70% residues, and 100% residues.
4) Grouped Di-Peptide/Tri-Peptide Composition (GDTPC). The 20 different amino acids can be categorized into five classes, including aliphatic group–*g1* (GAVLMI), aromatic group–*g2* (FYW), positively charged group–*g3* (KRH), negatively charged group–*g4* (DE), and uncharged group–*g5* (STCPNQ), according to their physicochemical properties, such as hydrophobicity, charge and molecular size of amino acids in a peptide sequence (Lee et al., 2011). In this study, the grouped di-peptide composition (GDPC) and the grouped tri-peptide composition (GTPC) are combined to present the feature vector in the peptide sequence. The GDPC is a variation of the di-peptide composition descriptor and can generate 25 descriptors (Chen et al., 2018; Chen et al., 2021). It is defined as follows:

$$f\left(i,j\right)=\;\frac{N_{ij}}{L-1},\;i,j\;\in \left\{g1,g2,g3,g4,g5\right\}$$

where $N_{ij}$ is the number of dipeptides coded by amino acid type groups i and j , and  L represents the length of a peptide sequence.

The GTPC is also a variation of the tri-peptide composition descriptor, and a total of 125 descriptors can be generated for a given peptide sequence (Chen et al., 2018; Chen et al., 2021). It is defined as follows:

$$f\left(i,j,s\right)=\;\frac{N_{ijs}}{L-1},\;i,j,s\;\in \left\{g1,g2,g3,g4,g5\right\}$$

where $N_{ijs}$ is the number of tripeptides coded by amino acid type groups i , j , and *s*, and  L represents the length of a peptide sequence.

### Feature Selection

Feature selection is an important process that can effectively reduce the number of redundant variables and the computational cost as well as solve overfitting problems in machine learning modeling. A variety of feature selection tools have been developed and applied to the identification of peptide sequences (Wang et al., 2013; Zou et al., 2016; Jung et al., 2019; He et al., 2020; Meng et al., 2020; Mostafa et al., 2020). For the first method applied to the optimal feature selection, we decided to utilize the MRMD tool (http://lab.malab.cn/soft/MRMD3.0/index.html) (Zou et al., 2016; He et al., 2020) following the PageRank strategy. MRMD is a feature ranking method based on function distance calculated by Pearson’s correlation coefficient to measure the independence of every feature and generates a sub-feature set with a low redundancy but strong relevance with the target class. The second method was the LGBM algorithm (Ke et al., 2017), which was used to select the best feature subsets based on the ranking of feature significance calculated by the LGBM classifier. Finally, the features selected by the MRMD and LGBM methods were used for modeling.

### Classifier Selection

In this study, eight popular machine learning algorithms were used, including Bagging, decision tree (DT), neighbors (KNN), light gradient boosting machine (LGBM), logistic regression (LR), GaussianNB (NB), random forest (RF) and support vector machines (SVM), to select a suitable algorithm for machine learning modeling. These algorithms are built into the scikit-learn toolkit package (Pedregosa et al., 2011), which can run in the *Python* program. Based on the feature selection matrix generated from the MRMD program, with regard to the eight algorithms, default hyperparameters were used for the initial evaluation during the process of classification performance. Additionally, we optimized the hyperparameters and selected the three most suitable classifiers, namely, RF, LGBM, and Bagging, for further comparative analysis. The best parameters were determined by grid search techniques, and the detailed settings are compiled in Supplementary Table S2.

### Performance Evaluation and Methods

For each predictive model, the quality was evaluated by measurement metrics for ten-fold cross-validation and an independent test method. In terms of measurement metrics, we used four standard evaluation metrics, namely, sensitivity (Se), specificity (Sp), accuracy (Acc), and Matthew correlation coefficient (MCC), to evaluate a model’s performance. These metrics were formulated as follows:

$$Se=\frac{TP}{TP+FN}\times 100\text{\%}$$

$$Sp=\frac{TN}{TN+FP}\times 100\%$$

$$Acc=\;\frac{TP+TN}{TP+TN+FN+FP}\times 100\text{\%}$$

$$MCC=\frac{TP\times TN-FP\times FN}{\sqrt{\left(TP+FN\right)\times \left(TP+FP\right)\times \left(TN+FP\right)\times \left(TN+FN\right)}}$$

where TP, TN, FP, and FN indicate the sample numbers of true positives, true negatives, false-positives, and false negatives, respectively. The Se of a test is also called the true positive rate and refers to the proportion of samples that are correctly classified as positive samples in the dataset among all real positive samples. The Sp of a test is also called the true negative rate and is the proportion of samples that are correctly classified as negative samples in the dataset among all real negative samples. Another two metrics, Acc and MCC, can comprehensively evaluate the performance of a predictor on balanced data. The Acc metric represents the ratio of a sample number of correct predictions to all numbers of input samples, but the MCC metric takes the ratio of positive and negative elements into account. Therefore, for unbalanced data, MCC would display a better predictive quality than Acc (Chicco and Jurman, 2020).

Additionally, the area under the receiver operating characteristic (auROC, or AUC) curve was introduced to evaluate the performance of a predictor. The auROC curve is plotted with a true positive rate on the *Y*-axis and the false-positive rate on the *X*-axis, with values ranging from 0 to 1. Having the auROC curve near the upper left or an auROC curve value = 1 reflects perfect prediction, while having an auROC curve value of 0.5 suggests random prediction of a model.

## Results and Discussion

### Analysis of Peptide Sequence Features

**FIGURE 2:**
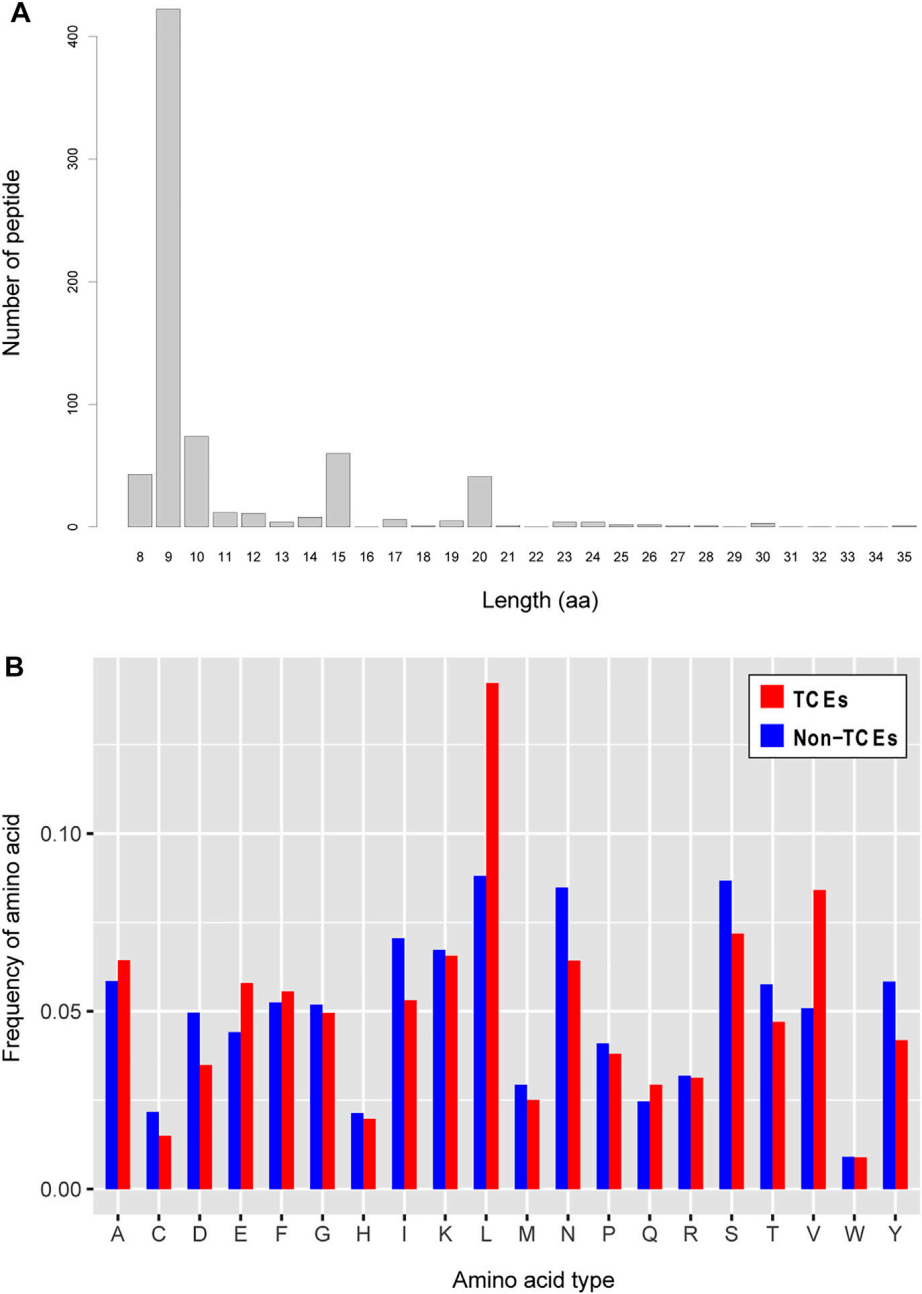
Analysis of amino acid sequence features. **(A)** Length distribution of the positive CD8^+^ T-cell epitopes. The horizontal axis represents the length of amino acids, and the vertical axis represents the number of epitopes in positive samples. **(B)** Distribution features of amino acid types with respect to the positive and negative CD8^+^ T-cell epitopes. The horizontal axis represents the twenty amino acids, and the vertical axis represents the occurrence frequency of an amino acid in all sequences.

In terms of peptide length, antigen epitopes that are presented to CD8^+^ T cells by MHC-I molecules are typical peptides between 8 and 11 amino acids in length, and occasionally a few noncanonical lengths overstep this range (Trolle et al., 2016). Additionally, the sequence characteristics of T-cell epitopes should largely reflect the specific binding ability to the MHC allele in the process of eliciting immune responses induced by pathogen infection. Motivated by these observations, we first investigated the length distribution of positive T-cell epitope sequences. The results are illustrated in Figure 2A, which shows the main distribution of sequence length is 9-mer peptides and that much longer peptides reach a length of up to 35 aa. As shown in Figure 2B, we also observed significant preferences in terms of amino acid appearance frequency between TCEs and non-TCEs, especially for leucine (L). Previous evidence has demonstrated that L is an important amino acid to mediate the adaptive immune response; specifically, L can play a role in T-cell activation and proliferation of immune cells (Ananieva et al., 2016). This implies that the preference of L in positive TCEs is essential feature information, in which the role of L not only serves as a biological activator of T-cell immunity but also may contribute to discriminating TCEs from non-TCEs.

### Initial Evaluation of a Single Feature Descriptor on Different Classifiers

In our scheme on feature learning, we evaluated the performance of individual feature descriptors by the utilization of eight extensively used machine learning classifiers, i.e., Bagging, DT, KNN, LGBM, LR, NB, RF, and SVM. These models were evaluated thoroughly by ten-fold cross-validation, and their performances were compared reciprocally. A detailed summary of these evaluation results is compiled in Supplementary Table S3, and the Acc values of all classifiers are shown in Table 1. Given that each descriptor has a fair comparison with the eight classifiers, as shown in Table 1, we noticed that in each feature descriptor, there were three classifiers, namely, Bagging, LGBM, and RF, that had better performances than other classifiers (the best Acc values are highlighted in bold font). In the results of ten-fold cross-validation, the LGBM classifier had the best performance on five feature descriptors (AAC, ASDC, CTDC, CTDT, and CTDD), followed by the RF classifier on three feature descriptors (AAC, GDPC, and GTPC); however, in the individual test result, there were four feature descriptors (CTDC, CTDT, CTDD, and GDPC) on the RF classifier that had the best performance, followed by two feature descriptors (ASDC and GTPC) on the LGBM classifier and one feature descriptor (AAC) on the Bagging classifier. Remarkably, the feature descriptor ASDC worked on the LGBM classifier and was able to obtain the best prediction results in both ten-fold cross-validation and the independent test, with Acc values of 75.443% and 76.761%, respectively. Therefore, the LGBM classifier can be chosen as the most suitable classifier for model deployment, if only a single feature descriptor is being considered.

**TABLE 1:** The accuracy (Acc) results of a single feature descriptor classified by different machine learning algorithms.

Feature descriptors		Classifiers and Acc values (%)							
		Bagging	DT	KNN	LGBM	LR	NB	RF	SVM
Ten-fold cross-validation	AAC	71.454	66.667	68.174	**73.670**	65.160	65.603	**73.670**	67.730
	ASDC	72.252	65.160	67.642	**75.443**	66.223	66.755	74.468	74.291
	CTDC	67.199	62.145	66.933	**68.528**	64.628	61.259	68.351	68.351
	CTDT	66.667	60.638	64.805	**67.996**	63.032	60.372	67.908	66.401
	CTDD	71.986	64.982	69.326	**75.089**	66.312	66.401	72.606	68.883
	GDPC	64.894	59.309	60.638	65.514	59.929	57.624	**66.223**	63.209
	GTPC	67.287	62.057	63.564	68.174	60.284	59.663	**68.528**	65.071
Independent test	AAC	**76.408**	67.606	73.592	76.056	66.901	65.493	74.648	67.606
	ASDC	75.352	65.845	69.366	**76.761**	67.254	69.014	75.704	74.296
	CTDC	72.535	63.028	66.197	70.070	67.254	63.380	**73.944**	71.479
	CTDT	65.493	60.915	65.141	65.141	66.197	60.211	**68.662**	64.085
	CTDD	69.014	62.324	66.197	72.183	68.662	66.901	**72.887**	70.775
	GDPC	63.028	50.704	59.859	64.789	61.268	61.972	**67.254**	65.141
	GTPC	66.197	59.859	65.141	**72.887**	63.380	63.028	68.662	66.197

The best Acc values to reflect the performance of different classifiers were highlighted in bold font.

### Comparison of Hybrid Multisource Features on Different Classifiers

Compared to machine learning techniques, in general, the sequence feature is a more critical element to achieve high accuracy in biological sequence classification, especially for the extensive applications of combining hybrid multisource features in machine learning modeling (Zhang et al., 2016; Mohan et al., 2019; Charoenkwan et al., 2020; Ao et al., 2021; Jiao et al., 2021). Based on the feature descriptors mentioned in Section 3.2, we combined similar feature types as a hybrid group, including CTDC + CTDT + CTDD (CCTD) and GDPC + GTPC (GDTPC), and the performances of four groups (AAC, ASDC, CCTD, and GDTPC) were compared thoroughly on the eight classifiers using ten-fold cross-validation. The detailed prediction results are summarized in Supplementary Table S2, and we reconfirmed that LGBM is a satisfactory classifier to differentiate TCEs from non-TCEs. To compare the performances of various hybrid features, as shown in Table 2, the prediction results of the LGBM classifier were generated based on the ten-fold cross-validation and independent test. Strikingly, the majority of prediction results of LGBM using hybrid features had an Acc value of more than 75%, which indicated that the prediction ability was greatly improved when compared to the single features. We also observed from Table 2 that the ten-fold cross-validation results of the AAC + ASDC + CCTD + GDTPC combination in particular, with metric values of 79.255% Acc, 0.585 MCC, 77.837% Se, and 80.674% Sp outperformed all the single or hybrid features, and therefore, this combination feature was selected for the subsequent analyses.

**TABLE 2:** The classification results of different hybrid feature combinations detected by the LGBM classifier.

Hybrid features	Ten-fold cross-validation				Independent test			
	Acc (%)	MCC	Se (%)	Sp (%)	Acc (%)	MCC	Se (%)	Sp (%)
AAC + ASDC + CCTD + GDTPC	**79.255**	**0.585**	**77.837**	**80.674**	**78.169**	**0.563**	**78.873**	77.465
ASDC + CCTD + GDTPC	78.103	0.562	76.596	79.610	76.056	0.521	77.465	74.648
AAC + ASDC + CCTD	77.660	0.553	76.064	79.255	77.113	0.542	76.056	78.169
AAC + CCTD + GDTPC	77.305	0.546	76.596	78.014	75.352	0.507	74.648	76.056
ASDC + CCTD	77.482	0.550	76.950	78.014	76.761	0.535	78.169	75.352
AAC + CCTD	76.684	0.534	75.887	77.482	75.352	0.507	76.056	74.648
ASDC + GDTPC	76.152	0.523	74.468	77.837	75.704	0.515	72.535	**78.873**
CCTD + GDTPC	76.064	0.522	74.468	77.660	77.465	0.550	**78.873**	76.056
AAC + ASDC	75.621	0.512	75.000	76.241	72.535	0.451	70.423	74.648
AAC + GDTPC	74.911	0.499	73.227	76.596	77.817	0.556	76.761	**78.873**
CCTD	75.621	0.513	74.645	76.596	74.648	0.495	**78.873**	70.423
GDTPC	69.592	0.392	68.262	70.922	72.535	0.451	71.831	73.239

The best metric values were highlighted in bold font.

### MRMD Serves as a Powerful Feature Selection Technology That Determines the Optimal Feature Space

Various feature selection technologies can be used for representation learning features. In the present study, we compared two feature selection technologies (MRMD and LGBM) by calculating the feature importance values, including the PageRank-based value for MRMD and Gini-based feature importance value for LGBM. Among the feature list results obtained by the two methods, we selected the top 520 features and employed the incremental feature selection (IFS) strategy to determine the optimal feature vector spaces, which are subsequently predicted on LGBM, RF, and Bagging classifiers.

**FIGURE 3:**
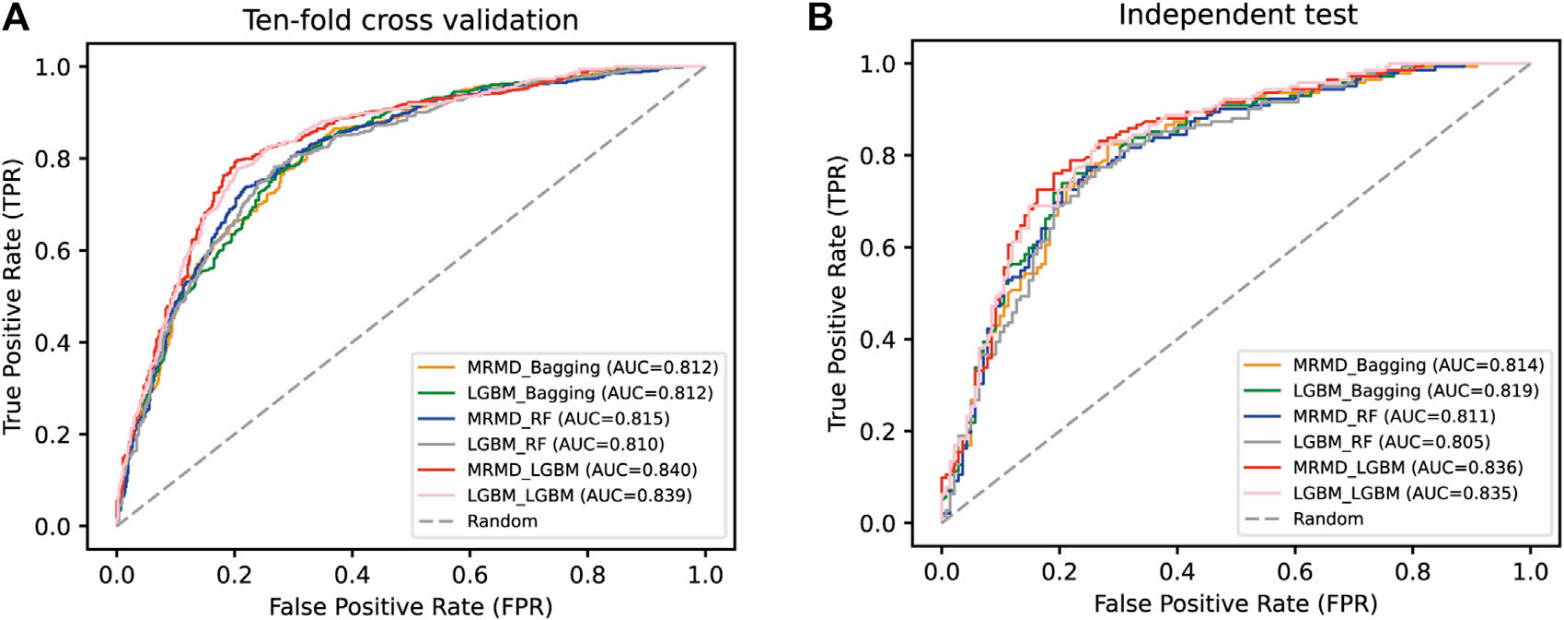
A comparison of the AUC curve in ten-fold cross-validation **(A)** and independent test **(B)**. Results were by a pairwise combination of two feature selection techniques (MRMD and LGBM) and three optimal classifiers (LGBM, RF, and Bagging).

As shown in Table 3, the ten-fold cross-validation results suggested that the MRMD + LGBM combination yielded the best prediction capability, with 79.255% Acc, 0.585 MCC, and 80.674% Sp, compared to the other five models (LGBM + LGBM, MRMD + RF, LGBM + RF, MRMD + Bagging, and LGBM + Bagging), except that the Se of MRMD + LGBM of 77.837% was lower than that of the LGBM + LGBM model; however, an independent test indicated that the MRMD + LGBM model was better than the other five combinations in all metrics. Furthermore, a separate AUC curve analysis is shown in Figure 3 and further illustrated that the MRMD + LGBM model with an AUC value of 0.840 in ten-fold cross-validation and an AUC value of 0.836 in the independent test outperformed the other five models.

**TABLE 3:** A comparison of classification results by a pairwise combination of two feature selection techniques (MRMD and LGBM) and three optimal classifiers (LGBM, RF, and Bagging).

Method	Ten-fold cross-validation				Independent test			
	Acc (%)	MCC	Se (%)	Sp (%)	Acc (%)	MCC	Se (%)	Sp (%)
MRMD + LGBM	**79.255**	**0.585**	77.837	**80.674**	**78.169**	**0.563**	**78.873**	**77.465**
LGBM + LGBM	78.457	0.569	**78.014**	78.901	77.113	0.542	77.465	76.761
MRMD + RF	75.887	0.518	73.404	78.369	74.648	0.493	72.535	76.761
LGBM + RF	75.355	0.507	74.645	76.064	74.648	0.493	73.944	75.352
MRMD + Bagging	73.316	0.466	72.163	74.468	75.352	0.507	73.239	77.465
LGBM + Bagging	74.202	0.484	72.695	75.709	75.704	0.514	76.056	75.352

The best metric values were highlighted in bold font.

**FIGURE 4:**
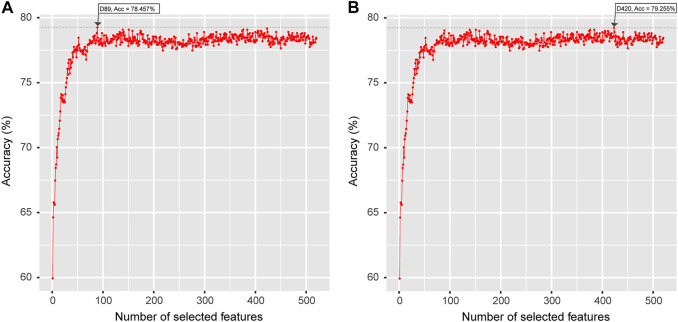
The optimal feature sets selected by LGBM feature importance ranking **(A)** and a well-established MRMD strategy **(B)**. The horizontal axis represents the number of selected features, and the vertical axis represents the accuracy value calculated by the LGBM classifier.

The optimal feature vector spaces detected by the IFS strategy suggested that the maximum accuracy of the LGBM + LGBM model was 78.457% with 89 features, which was less than the maximum accuracy of the MRMD + LGBM model of 79.255% with 420 features Figure 4. In the case of evaluating the computational cost of both models and considering the stability and robustness of the models, the MRMD + LGMB combination was finally selected as the best strategy for modeling and webserver development.

### User Guide of the Established Webserver

A user-friendly web server called CD8TCEI-EukPath was developed, and the users can freely enter the homepage *via* the link http://lab.malab.cn/∼hrs/CD8TCEI-EukPath/. The prediction interface can be accessed by clicking the “Prediction” or “CD8TCEI-EukPath” hyperlink, where the users can utilize amino acid sequences (FASTA format) to identify whether the input sequences are CD8TCEs or non-CD8TCEs. Briefly, the users should use short peptide sequences (generally 8–11 aa in length), paste the FASTA sequences in the left box, and click the “Submit” button for calculation. Immediately, the prediction results will be shown in the right box, which includes the protein name, predicted epitope (yes or no), and probability of belonging to CD8^+^ TCEs. If starting a new task, the users can click the “Resubmit” button and/or click the “Clear” button and paste new sequences to conduct computational predictions. Note that the computing resources of the webserver are limited for high-volume predictions, and the maximum number of sequences should be 1,000 at a time. In addition, using the established model, we also provided the prediction results of five important pathogen species (*Plasmodium*, *Toxoplasma*, *Trypanosoma*, *Leishmania*, and *Giardia*) based on the available mass spectrometry peptidome data obtained from the ProteomeXchange database (http://www.proteomexchange.org/). These prediction results can be downloaded freely from our web server and need to be further evaluated by MHC-peptide binding predictions and biological experiments.

### Conclusion

By comparing the performances of various single feature descriptors and hybrid feature descriptors using eight different classifiers, we selected a set of best features (AAC + ASDC + CCTD + GDTPC) and a satisfactory classifier (LGBM) for machine learning modeling. Following the state-of-the-art feature selection strategy of MRMD 3.0, we developed an effective sequence-based predictor named CD8TCEI-EukPath, capable of rapidly identifying eukaryotic pathogen-derived antigen epitopes for host CD8^+^ T cells from large peptide-coding datasets. As a first sequence-based predictor to identify T-cell epitopes in eukaryotic pathogens, CD8TCEI-EukPath achieved 79.255% Acc in ten-fold cross-validation and 78.169% Acc, 0.563 MCC, 78.873% Se and 77.465% Sp in the independent test. Meanwhile, a user-friendly web server was developed in the present work. We believe that this tool is helpful for scientists to evaluate the immunogenicity of a given peptide sequence before performing biological experiments. The current tool only applies to the identification of CD8^+^ T-cell epitopes in eukaryotic pathogens. In future works, we will apply deep representation learning features and state-of-the-art classification algorithms for CD4^+^ T-cell epitope and B-cell epitope prediction. By leveraging machine learning models to develop auxiliary tools, their combinations will assist in the development of peptide-based vaccines.

## Data Availability

The original contributions presented in the study are included in the article/Supplementary Material, further inquiries can be directed to the corresponding author/s.
