# Supplementary material for: CD8TCEI-EukPath: A Novel Predictor to Rapidly Identify CD8+ T-Cell Epitopes of Eukaryotic Pathogens Using a Hybrid Feature Selection Approach
Source: Front Genet. 2022 Jul 22;13:935989. doi: 10.3389/fgene.2022.935989 (PMC9354802; doi:10.3389/fgene.2022.935989)
Supplement: Supplementary file 1 [file DataSheet1.docx]

**Table S1.** Summary of positive and negative datasets used in this study.

| **Type** | **Species** | **Number of peptides** | **CD8^+^ T-cell epitope** | **Source** |
| --- | --- | --- | --- | --- |
| Positive datasets | *Plasmodium* | 359 | yes | IEDB database |
|  | *Trypanosoma* | 320 | yes | IEDB database |
|  | *Toxoplasma* | 53 | yes | IEDB database |
|  | *Fungi* | 47 | yes | IEDB database |
|  | *Piroplasma* | 29 | yes | IEDB database |
|  | *Giardia* | 1 | yes | IEDB database |
|  | *Trypanosoma* | 170 | yes | Reference (Ferragut et al., 2021) |
|  | *Plasmodium* | 130 | yes | Reference (Heide et al., 2019) |
|  | *Toxoplasma* | 71 | yes | Reference (Javadi Mamaghani et al., 2019) |
|  |  | Total=1,180 |  |  |
|  |  |  |  |  |
| Negative datasets | *Plasmodium* | 930 | no | IEDB database |
|  | *Trypanosoma* | 540 | no | IEDB database |
|  | *Toxoplasma* | 53 | no | IEDB database |
|  | *Fungi* | 105 | no | IEDB database |
|  | *Piroplasma* | 87 | no | IEDB database |
|  | *Giardia* | 0 | no | IEDB database |
|  |  | Total=1,715 |  |  |

**Table S2.** Summary of the hyperparameter settings used in this study.

| **Classifiers** | **Parameter** | **Value** |
| --- | --- | --- |
| Bagging | n_estimators | 20–180 with an interval of 20 |
| Decision Tree (DT) | max_depth | 2–100 with an interval of 2 |
| Kneighbors (KNN) | n_neighbors | 1-50 with an interval of 1 |
| Light Gradient Boosting Machine (LGBM) | n_estimators max_depth | 20–300 with an interval of 20 2–18 with an interval of 2 |
| Logistic Regression (LR) | Default parameters | / |
| GaussianNB (NB) | Default parameters | / |
| Random Forest (RF) | n_estimators max_depth | 20–180 with an interval of 20 2–18 with an interval of 2 |
| Support Vector Machine (SVM) | C gamma | 2^-5^–2^15^ with step size of 2 2^-15^–2^-5^ with step size of 2^-1^ |

**Table S3.** The classification results of single and hybrid features were calculated by eight classifiers.

| **Feature descriptors** | **Classifier** | **Acc** | **Se** | **Sp** | **MCC** |
| --- | --- | --- | --- | --- | --- |
| AAC | Bagging | 71.454 | 69.681 | 73.227 | 0.429 |
|  | DT | 66.667 | 62.411 | 70.922 | 0.335 |
|  | KNN | 68.174 | 68.262 | 68.085 | 0.363 |
|  | LGBM | **73.670** | **72.872** | 74.468 | 0.473 |
|  | LR | 65.160 | 64.007 | 66.312 | 0.303 |
|  | NB | 65.603 | 65.248 | 65.957 | 0.312 |
|  | RF | 73.670 | 71.099 | **76.241** | **0.474** |
|  | SVM | 67.730 | 68.085 | 67.376 | 0.355 |
| ASDC | Bagging | 72.252 | 70.922 | 73.582 | 0.445 |
|  | DT | 65.160 | **77.482** | 52.837 | 0.313 |
|  | KNN | 67.642 | 74.291 | 60.993 | 0.356 |
|  | LGBM | **75.443** | 73.936 | **76.950** | **0.509** |
|  | LR | 66.223 | 65.248 | 67.199 | 0.325 |
|  | NB | 66.755 | 66.844 | 66.667 | 0.335 |
|  | RF | 74.468 | 73.582 | 75.355 | 0.489 |
|  | SVM | 74.291 | 74.291 | 74.291 | 0.486 |
| CTDC | Bagging | 67.199 | 63.121 | **71.277** | 0.345 |
|  | DT | 62.145 | 61.525 | 62.766 | 0.243 |
|  | KNN | 66.933 | 63.652 | 70.213 | 0.339 |
|  | LGBM | **68.528** | **66.844** | 70.213 | **0.371** |
|  | LR | 64.628 | 63.652 | 65.603 | 0.293 |
|  | NB | 61.259 | 64.716 | 57.801 | 0.226 |
|  | RF | 68.351 | 66.667 | 70.035 | 0.367 |
|  | SVM | 68.351 | 68.262 | 68.440 | 0.367 |
| CTDT | Bagging | 66.667 | 64.184 | 69.149 | 0.334 |
|  | DT | 60.638 | 60.284 | 60.993 | 0.213 |
|  | KNN | 64.805 | 64.184 | 65.426 | 0.296 |
|  | LGBM | **67.996** | **66.489** | 69.504 | 0.360 |
|  | LR | 63.032 | 62.411 | 63.652 | 0.261 |
|  | NB | 60.372 | 56.738 | 64.007 | 0.208 |
|  | RF | 67.908 | 65.603 | 70.213 | **0.359** |
|  | SVM | 66.401 | 62.234 | **70.567** | 0.329 |
| CTDD | Bagging | 71.986 | 70.922 | 73.050 | 0.440 |
|  | DT | 64.982 | 62.766 | 67.199 | 0.300 |
|  | KNN | 69.326 | 66.312 | 72.340 | 0.387 |
|  | LGBM | **75.089** | **75.532** | **74.645** | **0.502** |
|  | LR | 66.312 | 65.603 | 67.021 | 0.326 |
|  | NB | 66.401 | 62.943 | 69.858 | 0.329 |
|  | RF | 72.606 | 71.277 | 73.936 | 0.452 |
|  | SVM | 68.883 | 65.957 | 71.809 | 0.378 |
| GDPC | Bagging | 64.894 | 61.348 | 68.440 | 0.299 |
|  | DT | 59.309 | 53.014 | 65.603 | 0.188 |
|  | KNN | 60.638 | 60.284 | 60.993 | 0.213 |
|  | LGBM | 65.514 | **63.475** | 67.553 | 0.311 |
|  | LR | 59.929 | 60.993 | 58.865 | 0.199 |
|  | NB | 57.624 | 57.801 | 57.447 | 0.152 |
|  | RF | **66.223** | 61.170 | **71.277** | **0.326** |
|  | SVM | 63.209 | 61.525 | 64.894 | 0.264 |
| GTPC | Bagging | 67.287 | 62.766 | 71.809 | 0.347 |
|  | DT | 62.057 | 59.929 | 64.184 | 0.241 |
|  | KNN | 63.564 | 63.475 | 63.652 | 0.271 |
|  | LGBM | 68.174 | 66.844 | 69.504 | 0.364 |
|  | LR | 60.284 | 59.752 | 60.816 | 0.206 |
|  | NB | 59.663 | 53.014 | 66.312 | 0.195 |
|  | RF | **68.528** | 63.475 | **73.582** | **0.372** |
|  | SVM | 65.071 | **67.908** | 62.234 | 0.302 |
| CTDC + CTDT + CTDD (CCTD) | Bagging | 70.390 | 69.858 | 70.922 | 0.408 |
|  | DT | 66.135 | 59.043 | 73.227 | 0.326 |
|  | KNN | 68.262 | 65.426 | 71.099 | 0.366 |
|  | LGBM | **75.621** | **74.645** | **76.596** | **0.513** |
|  | LR | 68.351 | 68.262 | 68.440 | 0.367 |
|  | NB | 66.135 | 65.603 | 66.667 | 0.323 |
|  | RF | 72.429 | 71.099 | 73.759 | 0.449 |
|  | SVM | 70.301 | 67.908 | 72.695 | 0.406 |
| GDPC + GTPC (GDTPC) | Bagging | 66.489 | 61.879 | 71.099 | 0.331 |
|  | DT | 60.550 | 60.461 | 60.638 | 0.211 |
|  | KNN | 63.032 | 63.121 | 62.943 | 0.261 |
|  | LGBM | **69.592** | **68.262** | 70.922 | 0.392 |
|  | LR | 60.816 | 62.234 | 59.397 | 0.216 |
|  | NB | 59.486 | 48.404 | 70.567 | 0.195 |
|  | RF | 68.794 | 64.716 | **72.872** | **0.377** |
|  | SVM | 65.514 | 64.716 | 66.312 | 0.310 |
| AAC+ASDC | Bagging | 72.606 | 70.922 | 74.291 | 0.452 |
|  | DT | 65.869 | 65.248 | 66.489 | 0.317 |
|  | KNN | 66.401 | 70.213 | 62.589 | 0.329 |
|  | LGBM | **75.621** | **75.000** | **76.241** | **0.512** |
|  | LR | 67.819 | 67.553 | 68.085 | 0.356 |
|  | NB | 68.085 | 73.227 | 62.943 | 0.364 |
|  | RF | 74.468 | 73.404 | 75.532 | 0.489 |
|  | SVM | 73.493 | 73.050 | 73.936 | 0.470 |
| AAC+CCTD | Bagging | 71.631 | 70.213 | 73.050 | 0.433 |
|  | DT | 64.628 | 59.043 | 70.213 | 0.294 |
|  | KNN | 69.504 | 69.149 | 69.858 | 0.390 |
|  | LGBM | **76.684** | **75.887** | **77.482** | **0.534** |
|  | LR | 69.326 | 68.617 | 70.035 | 0.387 |
|  | NB | 66.135 | 68.440 | 63.830 | 0.323 |
|  | RF | 74.025 | 72.340 | 75.709 | 0.481 |
|  | SVM | 70.833 | 68.262 | 73.404 | 0.417 |
| AAC+GDTPC | Bagging | 73.316 | 70.035 | 76.596 | **0.467** |
|  | DT | 63.564 | 61.702 | 65.426 | 0.271 |
|  | KNN | 66.489 | 66.135 | 66.844 | 0.330 |
|  | LGBM | **74.911** | **73.227** | 76.596 | 0.499 |
|  | LR | 66.489 | 66.489 | 66.489 | 0.330 |
|  | NB | 61.968 | 65.426 | 58.511 | 0.240 |
|  | RF | 73.759 | 70.390 | **77.128** | **0.476** |
|  | SVM | 70.035 | 69.858 | 70.213 | 0.401 |
| ASDC+CCTD | Bagging | 74.025 | 71.986 | 76.064 | 0.481 |
|  | DT | 64.894 | 56.383 | 73.404 | 0.302 |
|  | KNN | 70.833 | **83.156** | 58.511 | 0.430 |
|  | LGBM | **77.482** | 76.950 | **78.014** | **0.550** |
|  | LR | 65.957 | 65.248 | 66.667 | 0.319 |
|  | NB | 68.617 | 69.149 | 68.085 | 0.372 |
|  | RF | 74.113 | 73.759 | 74.468 | 0.482 |
|  | SVM | 73.227 | 72.872 | 73.582 | 0.465 |
| ASDC+GDTPC | Bagging | 74.645 | 72.340 | 76.950 | 0.493 |
|  | DT | 64.716 | 73.936 | 55.496 | 0.299 |
|  | KNN | 68.440 | 69.681 | 67.199 | 0.369 |
|  | LGBM | **76.152** | **74.468** | **77.837** | **0.523** |
|  | LR | 66.844 | 65.071 | 68.617 | 0.337 |
|  | NB | 67.908 | 67.908 | 67.908 | 0.358 |
|  | RF | 73.848 | 71.277 | 76.418 | 0.478 |
|  | SVM | 75.089 | **74.468** | 75.709 | 0.502 |
| CCTD+GDTPC | Bagging | 71.011 | 68.794 | 73.227 | 0.421 |
|  | DT | 64.894 | 64.716 | 65.071 | 0.298 |
|  | KNN | 67.553 | 58.688 | 76.418 | 0.357 |
|  | LGBM | **76.064** | **74.468** | **77.660** | **0.522** |
|  | LR | 67.553 | 67.730 | 67.376 | 0.351 |
|  | NB | 65.248 | 66.844 | 63.652 | 0.305 |
|  | RF | 74.291 | 73.227 | 75.355 | 0.486 |
|  | SVM | 71.011 | 68.085 | 73.936 | 0.421 |
| AAC+ASDC+CCTD | Bagging | 72.074 | 70.745 | 73.404 | 0.442 |
|  | DT | 63.741 | 60.638 | 66.844 | 0.275 |
|  | KNN | 70.035 | **80.142** | 59.929 | 0.409 |
|  | LGBM | **77.660** | 76.064 | **79.255** | **0.553** |
|  | LR | 66.223 | 65.426 | 67.021 | 0.325 |
|  | NB | 68.440 | 70.567 | 66.312 | 0.369 |
|  | RF | 74.025 | 72.695 | 75.355 | 0.481 |
|  | SVM | 73.582 | 72.518 | 74.645 | 0.472 |
| AAC+CCTD+GDTPC | Bagging | 72.340 | 69.149 | 75.532 | 0.448 |
|  | DT | 64.628 | 59.043 | 70.213 | 0.294 |
|  | KNN | 70.213 | 69.326 | 71.099 | 0.404 |
|  | LGBM | **77.305** | **76.596** | **78.014** | **0.546** |
|  | LR | 67.642 | 65.957 | 69.326 | 0.353 |
|  | NB | 67.642 | 65.780 | 69.504 | 0.353 |
|  | RF | 75.000 | 73.582 | 76.418 | 0.500 |
|  | SVM | 71.986 | 71.277 | 72.695 | 0.440 |
| ASDC+CCTD+GDTPC | Bagging | 73.227 | 72.340 | 74.113 | 0.465 |
|  | DT | 64.894 | 57.624 | 72.163 | 0.301 |
|  | KNN | 70.035 | 60.461 | **79.610** | 0.408 |
|  | LGBM | **78.103** | **76.596** | **79.610** | **0.562** |
|  | LR | 66.312 | 66.135 | 66.489 | 0.326 |
|  | NB | 69.504 | 69.326 | 69.681 | 0.390 |
|  | RF | 74.734 | 74.291 | 75.177 | 0.495 |
|  | SVM | 74.025 | 73.404 | 74.645 | 0.481 |
| AAC+ASDC+CCTD+GDTPC | Bagging | 73.316 | 72.163 | 74.468 | 0.466 |
|  | DT | 65.160 | 61.702 | 68.617 | 0.304 |
|  | KNN | 71.365 | 72.340 | 70.390 | 0.427 |
|  | LGBM | **79.255** | 77.837 | **80.674** | **0.585** |
|  | LR | 66.755 | 65.426 | 68.085 | 0.335 |
|  | NB | 69.238 | 68.440 | 70.035 | 0.385 |
|  | RF | 75.887 | 73.404 | 78.369 | 0.518 |
|  | SVM | 74.823 | **78.014** | 71.631 | 0.497 |

The best Acc values to reflect the performance of different classifiers were highlighted in red bold font.

**Appendix:**

AAC: Amino Acid Composition

ASDC: Adaptive Skip Dinucleotide Composition

CTDC: Composition/Transition/Distribution for Composition

CTDT: Composition/Transition/Distribution for Transition

CTDD: Composition/Transition/Distribution for Distribution

CCTD: CTDC + CTDT + CTDD

GDPC: Grouped Dipeptide Composition

GTPC: Grouped Tripeptide Composition

GDTPC: GDPC + GTPC
